# Supplementary material for: Structural insights into translocation and tailored synthesis of hyaluronan
Source: Nat Struct Mol Biol. 2024 Sep 25;32(1):161–71. doi: 10.1038/s41594-024-01389-1 (PMC11750622; doi:10.1038/s41594-024-01389-1)
Supplement: Supplementary file 1 — Supplementary Table 1, Fig. 1 and discussion. [file 41594_2024_1389_MOESM1_ESM.pdf]

nature structural & molecular biology

Supplementary information

https://doi.org/10.1038/s41594-024-01389-1

Structural insights into translocation and tailored synthesis of hyaluronan

In the format provided by the authors and unedited

Supplementary Table 1 Oligos used to generate all XlHAS1 and CvHAS mutants in this study.

xHAS1_H72A_F	AATGCTTCTCgcCCTGATGATGCAGAG
xHAS1_H72A_R	GCAAGACCATAAAGCCCA
xHAS1_H72F_F	ggtcttgcaatgcttctcttcctgatgatgcagagcct
xHAS1_H72F_R	aggctctgcatcatcaggaagagaagcattgcaagacc
xHAS_K218A_F	atgcaacagtggggcggagcaagagaggtcatgtacac
xHAS_K218A_R	gtgtacatgacctctcttgctccgccccactgttgcat
xHAS_R287A_F	gttaaacgccatccagtaagccaggctgctcatgaaacta
xHAS_R287A_R	tagtttcatgagcagcctggcttactggatggcgtttaac
xHAS_R296A_F	TAACGTGGAGgcGGCCTGCCAG
xHAS_R296A_R	AACGCCATCCAGTAACGC
xHAS1_C307A_F	acttcgactgcgtgtccgctataagtggacctctgg
xHAS1_C307A_R	ccagaggtccacttatagcggacacgcagtcgaagt
xHAS1_C307S_F	cttcgactgcgtgtccagtataagtggacctct
xHAS1_C307S_R	agaggtccacttatactggacacgcagtcgaag
xHAS1_C337A_F	agacagaaatttttgggaacctatgctactttgggagatgatagacatc
xHAS1_C337A_R	gatgtctatcatctcccaaagtagcataggttcccaaaaatttctgtct
xHAS1_C337S_F	acagaaatttttgggaacctatagtactttgggagatgatagac
xHAS1_C337S_R	gtctatcatctcccaaagtactataggttcccaaaaatttctgt
xHAS_R381A_F	CCAGCAAACCgcGTGGACCAAG
xHAS_R381A_R	TTCAACCACCGGAGATAC
xHAS_K448A_F	GTCTCTCTTCgcATCCATCTATGCC
xHAS_K448A_R	ATGATCTGGATGCACAGG
xHAS1_W491A_F	gaccttaaacaagaccggtgcgggaacatctgggcg
xHAS1_W491A_R	cgcccagatgttcccgcaccggtcttgtttaaggtc
xHAS1_W491F_F	accttaaacaagaccggtttcggaacatctgggcgc
xHAS1_W491F_R	gcgcccagatgttccgaaaccggtcttgtttaaggt
xHAS1_T493A_F	gaccggttggggagcatctgggcgcaa
xHAS1_T493A_R	ttgcgcccagatgctccccaaccggtc
xHAS1_T493S_F	gaccggttggggatcatctgggcgcaa
xHAS1_T493S_R	ttgcgcccagatgatccccaaccggtc
xHAS_K218R_F	gcaacagtggggcggaagaagagaggtcatgtac
xHAS_K218R_R	gtacatgacctctcttcttccgccccactgttgc
xHAS_R287K_F	GAGCAGCCTGaagTACTGGATGG
xHAS_R287K_R	ATGAAACTAATGAAGGAATCATAAG
xHAS_R296K_F	atggcgtttaacgtggagaaggcctgccag
xHAS_R296K_R	ctggcaggccttctccacgttaaacgccat
xHAS_R381K_F	gtggttgaaccagcaaaccaagtggaccaagtcctacttc
xHAS_R381K_R	gaagtaggacttggtccacttggtttgctggttcaaccac
xHAS_R448R_F	agatcatgtctctcttcagatccatctatgcctgctg
xHAS_R448R_R	cagcaggcatagatggatctgaagagagacatgatct
xHAS1_R496A_F	AACATCTGGGgcgAAGAAGATAGTAGGCAAC
xHAS1_R496A_R	CCCCAACCGGTCTTG
xHAS1_R496K_F	AACATCTGGGaaaAAGAAGATAGTAGGC
xHAS1_R496K_R	CCCCAACCGGTCTTG
CvHAS_G455A_F	GACATTGCTTGGGCCACTCGTGGTG
CvHAS_G455A_R	CACCACGAGTGGCCCAAGCAATGTC
CvHAS_W454A_F	GTTTGACATTGCTGCGGGCACTCGTG
CvHAS_W454A_R	CACGAGTGCCCGCAGCAATGTCAAAC
CvHAS_R457K_F	CATTGCTTGGGGCACTAAAGGTGGCAACGCC
CvHAS_R457K_R	GGCGTTGCCACCTTTAGTGCCCCAAGCAATG
CvHAS_W454F_F	GTTTGACATTGCTTTCGGCACTCGTG
CvHAS_W454F_R	CACGAGTGCCGAAAGCAATGTCAAAC

Supplementary Figure 1 Model of HA biosynthesis and translocation.

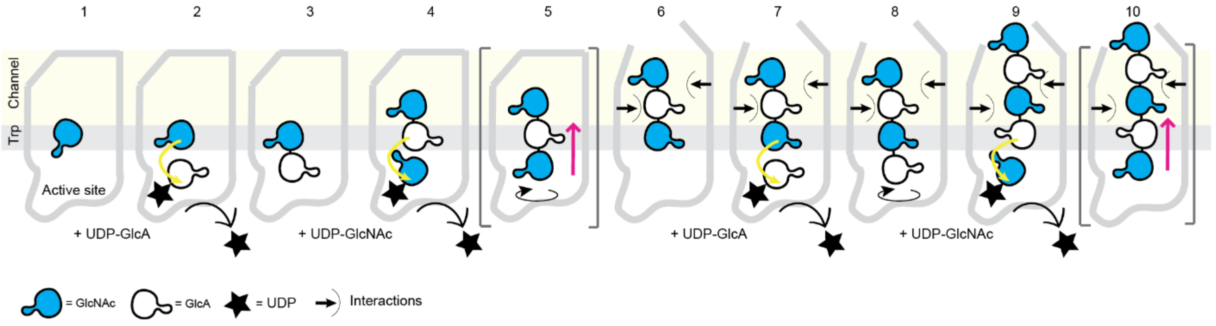

Supplementary Discussion on HA biosynthesis and translocation.

Predicted solution and experimentally determined high resolution structures of HA suggest a preferred orientation of the disaccharide's GlcA and GlcNAc units where the carboxylate and acetamido groups point roughly in the same direction. Specifically, the carboxylate group of the glucuronic acid unit at the disaccharide's reducing end is in close proximity (but not necessarily in hydrogen bonding distance) to GlcNAc's acetamido nitrogen at the disaccharide's non-reducing end. Often, this configuration is stabilized by a hydrogen bond between the ring oxygen of the non-reducing end sugar with the C3 or C4 hydroxyl (for GlcA and GlcNAc, respectively) of the sugar unit at the reducing end of the repeat unit. Within a polymer, the disaccharide repeats can rotate around the glycosidic bonds and particular configurations can be stabilized by interactions with proteinaceous receptors. These HA properties likely affect its coordination, elongation, and translocation by HAS.

Experimental structures of primed HAS reveal that the priming GlcNAc unit is coordinated in a specific binding pose with its acetamido group pointing away from the base catalyst (for simplicity, this is referred to as 'pointing left'). Extending the primer with GlcA at its C3 hydroxyl (i.e. GlcA being at the non-reducing end) creates a HA disaccharide with the acetamido and carboxylate groups pointing in opposite directions and both glycopyranose rings being roughly co-planar. Next, adding a GlcNAc moiety to the C4 hydroxyl of the GlcA unit creates a trisaccharide. Based on the observations described above, this terminal GlcNAc unit then likely rotates around the newly formed glycosidic bond, such that its acetamido group points 'right' (towards the base catalyst), in the same direction as the carboxylate of the preceding GlcA unit. In this configuration, all three glycopyranose rings would, approximately, be in the same plane. Translocating this trisaccharide by one sugar unit prior to another elongation reaction places the GlcNAc unit at the acceptor site in the opposite direction as observed for the priming GlcNAc unit (with its acetamido group pointing 'right'). While a GlcNAc monosaccharide primer does not seem to be stably bound in this orientation, a GlcNAc acceptor within a polymer may be stabilized in this orientation by the polymer itself. Similar polymer-stabilized acceptor poses may exist in cellulose and chitin, which are homopolysaccharides of glucose and GlcNAc, respectively.

Adding a GlcA unit to this GlcNAc-ending trisaccharide likely favors its rotation around the newly formed connection. This places its carboxylate group to the ‘left’ and aligns the plane of its glycopyranose ring with the preceding polymer, thereby facilitating translocation.

Based on this model, the HA disaccharide repeat units enter the TM channel in two different orientations with the carboxylate and acetamido substituents either pointing ‘left’ or ‘right’. Accordingly, the polymer density of our HA-bound HAS structure likely represents an ensemble average of two different polymer orientations. However, we cannot exclude that one polymer pose is advantageous for *in vitro* structural analysis, thereby explaining the apparently better fit of the modeled orientation with the EM map.
