# Supplementary material for: Structural insights into translocation and tailored synthesis of hyaluronan
Source: Nat Struct Mol Biol. 2024 Sep 25;32(1):161–71. doi: 10.1038/s41594-024-01389-1 (PMC11750622; doi:10.1038/s41594-024-01389-1)
Supplement: Supplementary file 2 — Reporting Summary [file 41594_2024_1389_MOESM2_ESM.pdf]

nature portfolio

Corresponding author(s): Jochen Zimmer, PhD

Last updated by author(s): Jun 17, 2024

Reporting Summary

Nature Portfolio wishes to improve the reproducibility of the work that we publish. This form provides structure for consistency and transparency in reporting. For further information on Nature Portfolio policies, see our Editorial Policies and the Editorial Policy Checklist.

Statistics

For all statistical analyses, confirm that the following items are present in the figure legend, table legend, main text, or Methods section.

n/a	Confirmed	
☐	☒	The exact sample size (*n*) for each experimental group/condition, given as a discrete number and unit of measurement
☐	☒	A statement on whether measurements were taken from distinct samples or whether the same sample was measured repeatedly
☒	☐	The statistical test(s) used AND whether they are one- or two-sided<br>*Only common tests should be described solely by name; describe more complex techniques in the Methods section.*
☒	☐	A description of all covariates tested
☒	☐	A description of any assumptions or corrections, such as tests of normality and adjustment for multiple comparisons
☐	☒	A full description of the statistical parameters including central tendency (e.g. means) or other basic estimates (e.g. regression coefficient) AND variation (e.g. standard deviation) or associated estimates of uncertainty (e.g. confidence intervals)
☒	☐	For null hypothesis testing, the test statistic (e.g. *F*, *t*, *r*) with confidence intervals, effect sizes, degrees of freedom and *P* value noted<br>*Give P values as exact values whenever suitable.*
☒	☐	For Bayesian analysis, information on the choice of priors and Markov chain Monte Carlo settings
☒	☐	For hierarchical and complex designs, identification of the appropriate level for tests and full reporting of outcomes
☒	☐	Estimates of effect sizes (e.g. Cohen's *d*, Pearson's *r*), indicating how they were calculated

Our web collection on statistics for biologists contains articles on many of the points above.

Software and code

Policy information about availability of computer code

Data collection	EPU 2.5 (ThermoFisher) was used to collect cryoEM data. SoftMax 7.2 (MolecularDevices) software was used to record UDP release curves.
Data analysis	CryoEM data was processed in cryoSPARC 4.4 and analyzed in Chimera 1.17.1. Model refinement was performed in Phenix 1.21 and WinCoot 0.9.8.93. ChimeraX 1.6 and PyMOL 2.5.5 were used for cryoEM and model visualization. Catalytic activity data was processed in MS Excel 365 and GraphPad Prism 6.

For manuscripts utilizing custom algorithms or software that are central to the research but not yet described in published literature, software must be made available to editors and reviewers. We strongly encourage code deposition in a community repository (e.g. GitHub). See the Nature Portfolio guidelines for submitting code & software for further information.

Data

Policy information about availability of data

All manuscripts must include a data availability statement. This statement should provide the following information, where applicable:

- Accession codes, unique identifiers, or web links for publicly available datasets
- A description of any restrictions on data availability
- For clinical datasets or third party data, please ensure that the statement adheres to our policy

Previously determined structure of CvHAS (PDB 7SP6) was used to build CvHAS models determined in this study. XlHAS1 Alphafold2 prediction (B1WB39) was used to build XlHAS1 apo model. Coordinates and EM maps have been deposited at the Protein Data Bank and Electron Microscopy Data Bank under accession codes

8SMM/EMD-40591, 8SMN/EMD-40594, 8SMP/EMD-40598, 8SND/EMD-40623, 8SNC/EMD-40622, 8SNE/EMD-40624 for Xl-HAS-1 apo, Xl-HAS-1 HA-bound, Xl-HAS-1 UDP-bound, Cv-HAS GlcNAc and UDP-GlcA-bound, Cv-HAS GlcA-extended GlcNAc-bound, Cv-HAS GlcA-extended GlcNAc and UDP-bound, respectively.

Research involving human participants, their data, or biological material

Policy information about studies with human participants or human data. See also policy information about sex, gender (identity/presentation), and sexual orientation and race, ethnicity and racism.

Reporting on sex and gender	n/a
Reporting on race, ethnicity, or other socially relevant groupings	n/a
Population characteristics	n/a
Recruitment	n/a
Ethics oversight	n/a

Note that full information on the approval of the study protocol must also be provided in the manuscript.

Field-specific reporting

Please select the one below that is the best fit for your research. If you are not sure, read the appropriate sections before making your selection.

☒ Life sciences ☐ Behavioural & social sciences ☐ Ecological, evolutionary & environmental sciences

For a reference copy of the document with all sections, see nature.com/documents/nr-reporting-summary-flat.pdf

Life sciences study design

All studies must disclose on these points even when the disclosure is negative.

Sample size	No sample size calculation was performed as the experiments were performed in vitro and the data was reproducible. Each activity assay was performed in at least three technical replicates to calculate standard deviations.
Data exclusions	No biochemical data was excluded. Low quality cryoEM datasets were excluded.
Replication	Each activity assay was performed in biological triplicates or quadruplicates and each experiment was repeated at least three times. Multiple cryoEM datasets on multiple samples were collected for the individual HAS states. Datasets resulting in the highest quality maps are reported in this study.
Randomization	Not applicable as this is not a clinical study.
Blinding	Not applicable as this is not a clinical study.

Reporting for specific materials, systems and methods

We require information from authors about some types of materials, experimental systems and methods used in many studies. Here, indicate whether each material, system or method listed is relevant to your study. If you are not sure if a list item applies to your research, read the appropriate section before selecting a response.

Materials & experimental systems

n/a	Involved in the study	
☐	☒	Antibodies
☐	☒	Eukaryotic cell lines
☒	☐	Palaeontology and archaeology
☒	☐	Animals and other organisms
☒	☐	Clinical data
☒	☐	Dual use research of concern
☒	☐	Plants

Methods

n/a	Involved in the study	
☒	☐	ChIP-seq
☒	☐	Flow cytometry
☒	☐	MRI-based neuroimaging

Antibodies

Antibodies used	Commercially available primary anti-His (Qiagen, cat. # 34660, diluted 1:1000) and secondary DyLight800-conjugated anti-Mouse (Thomas Scientific, cat. # 610-145-002-0.5, diluted 1:10000) antibodies for Western Blots.
Validation	From manufacturer, see https://www.qiagen.com/us/products/discovery-and-translational-research/protein-purification/taggedprotein-expression-purification-detection/anti-his-antibodies-bsa-free?catno=34660

Eukaryotic cell lines

Policy information about cell lines and Sex and Gender in Research

Cell line source(s)	Spodoptera frugiperda (Sf9) insect cells were purchased from Expression Systems.
Authentication	Non authenticated.
Mycoplasma contamination	Not tested for mycoplasma contamination.
Commonly misidentified lines (See ICLAC register)	None used.

Plants

Seed stocks	n/a
Novel plant genotypes	n/a
Authentication	n/a
