# Supplementary figures and images for: Structural insights into translocation and tailored synthesis of hyaluronan
Source: Nat Struct Mol Biol. 2024 Sep 25;32(1):161–71. doi: 10.1038/s41594-024-01389-1 (PMC11750622; doi:10.1038/s41594-024-01389-1)

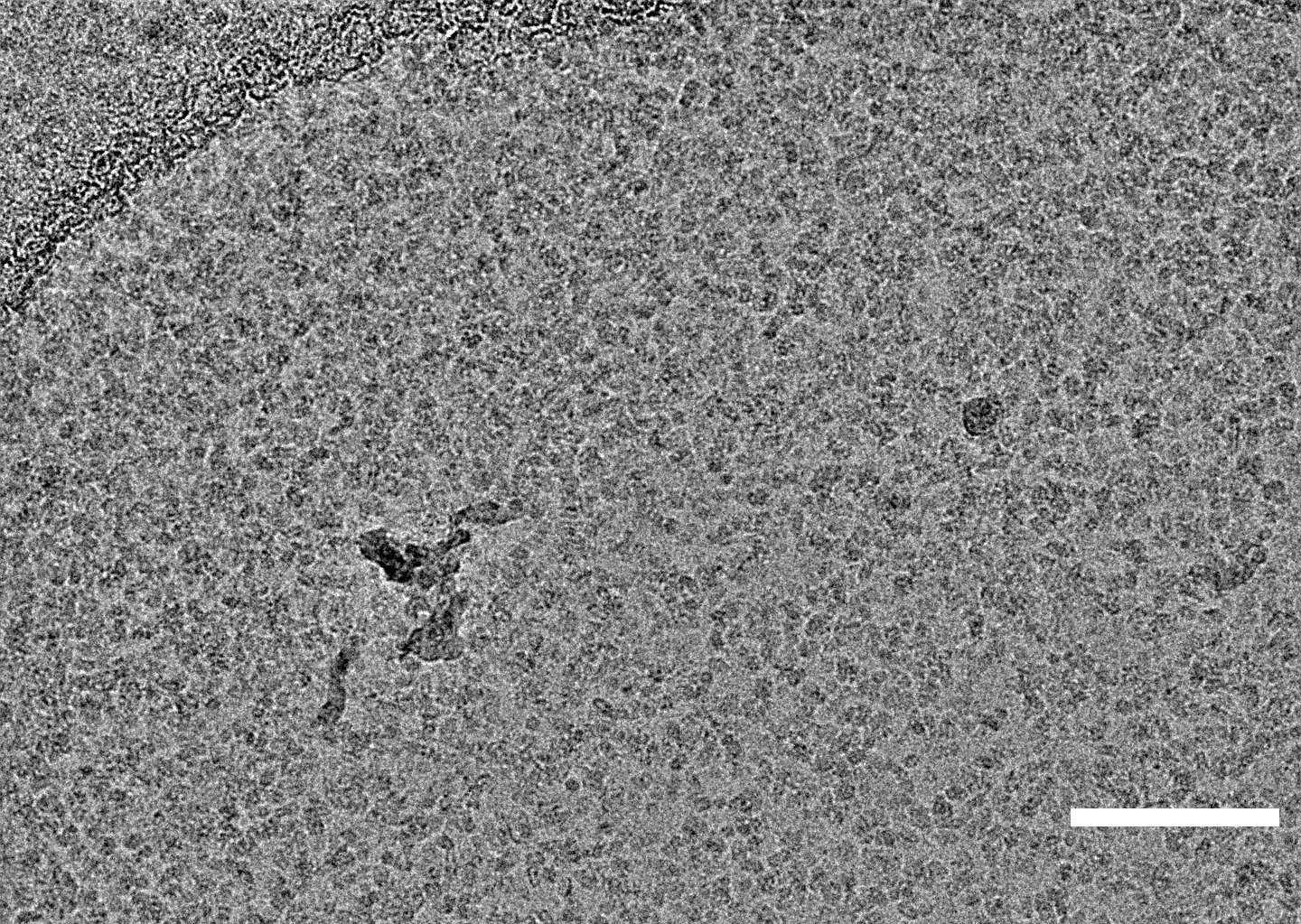

Supplement: Supplementary file 4 — Representative cryo-EM micrograph for XlHAS1 apo dataset. [file 41594_2024_1389_MOESM4_ESM.jpg]

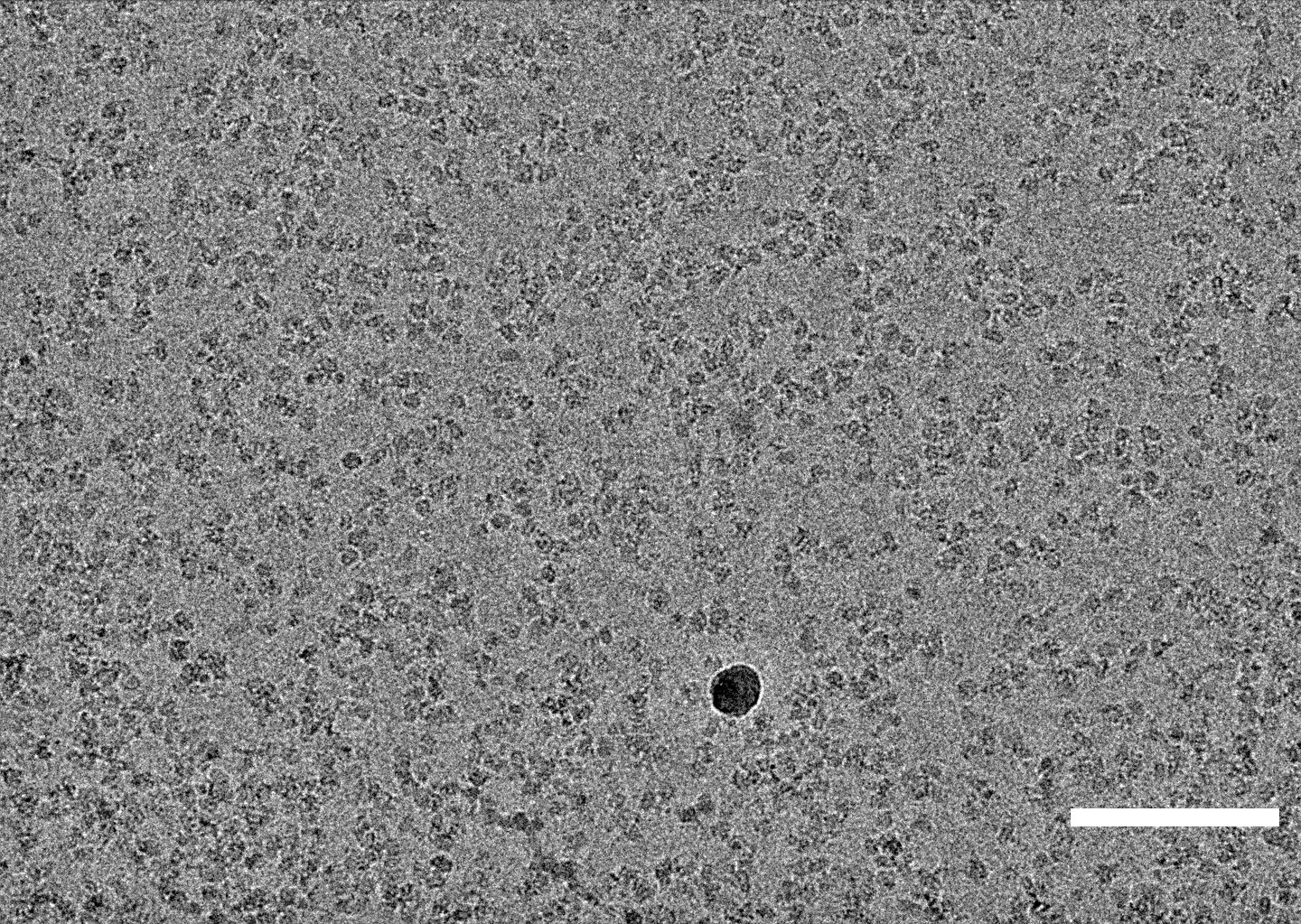

Supplement: Supplementary file 5 — Representative cryo-EM micrograph for XlHAS1 HA-bound dataset. [file 41594_2024_1389_MOESM5_ESM.jpg]

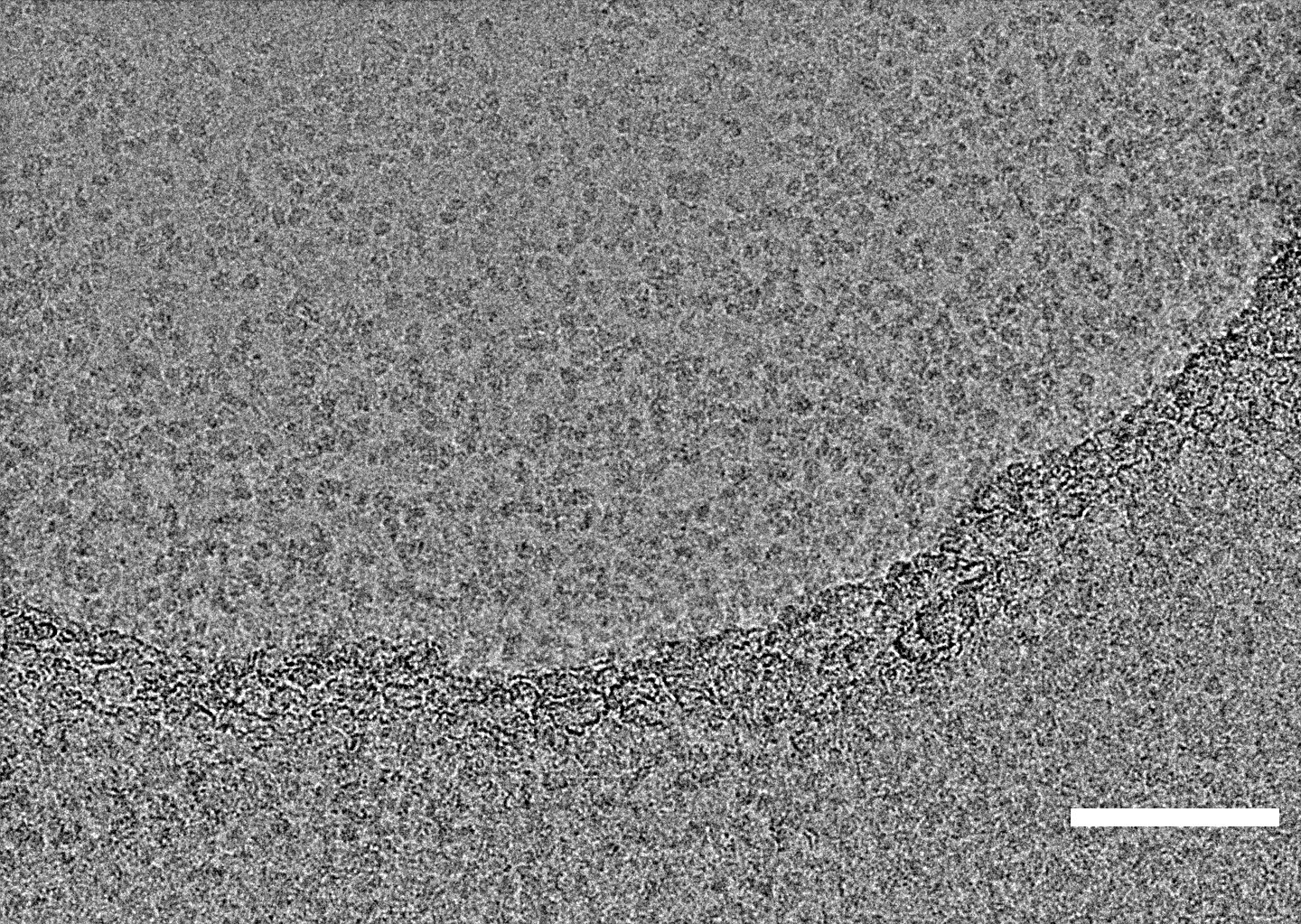

Supplement: Supplementary file 6 — Representative cryo-EM micrograph for XlHAS1 UDP-bound dataset. [file 41594_2024_1389_MOESM6_ESM.jpg]

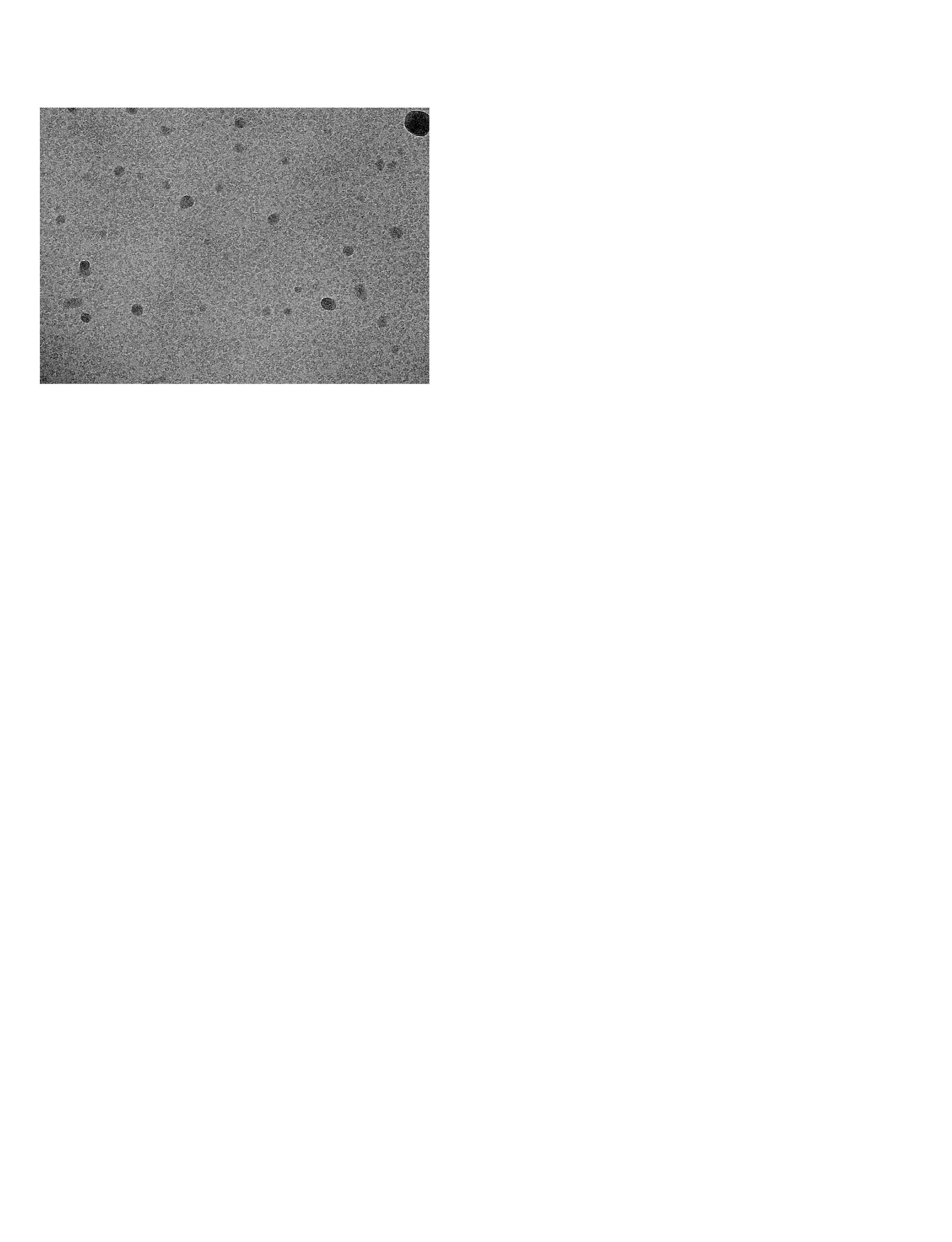

Supplement: Supplementary file 7 — Representative cryo-EM micrograph for CvHAS primed and UDP-GlcA-bound dataset. [file 41594_2024_1389_MOESM7_ESM.jpg]

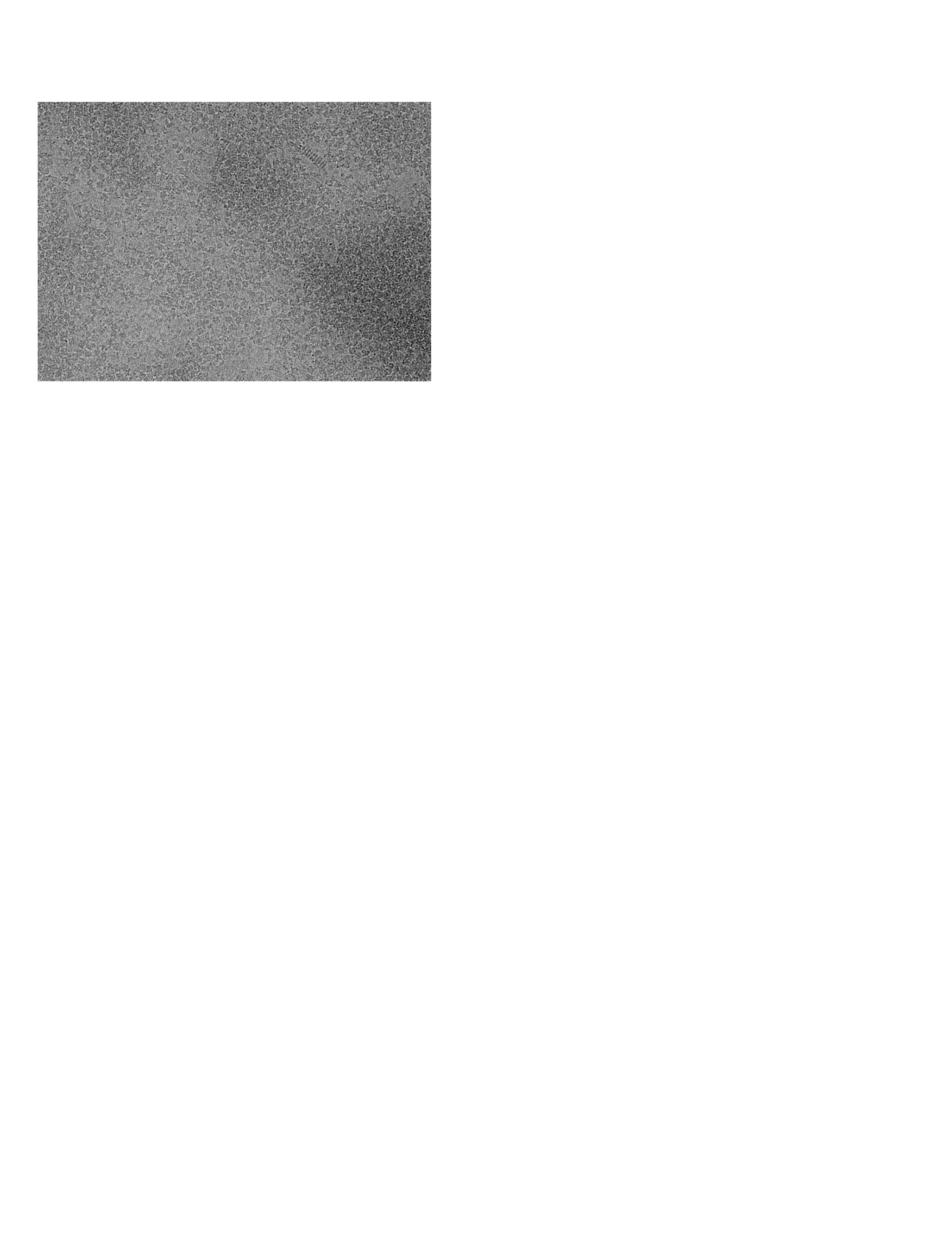

Supplement: Supplementary file 8 — Representative cryo-EM micrograph for CvHAS HA2-bound dataset. [file 41594_2024_1389_MOESM8_ESM.jpg]

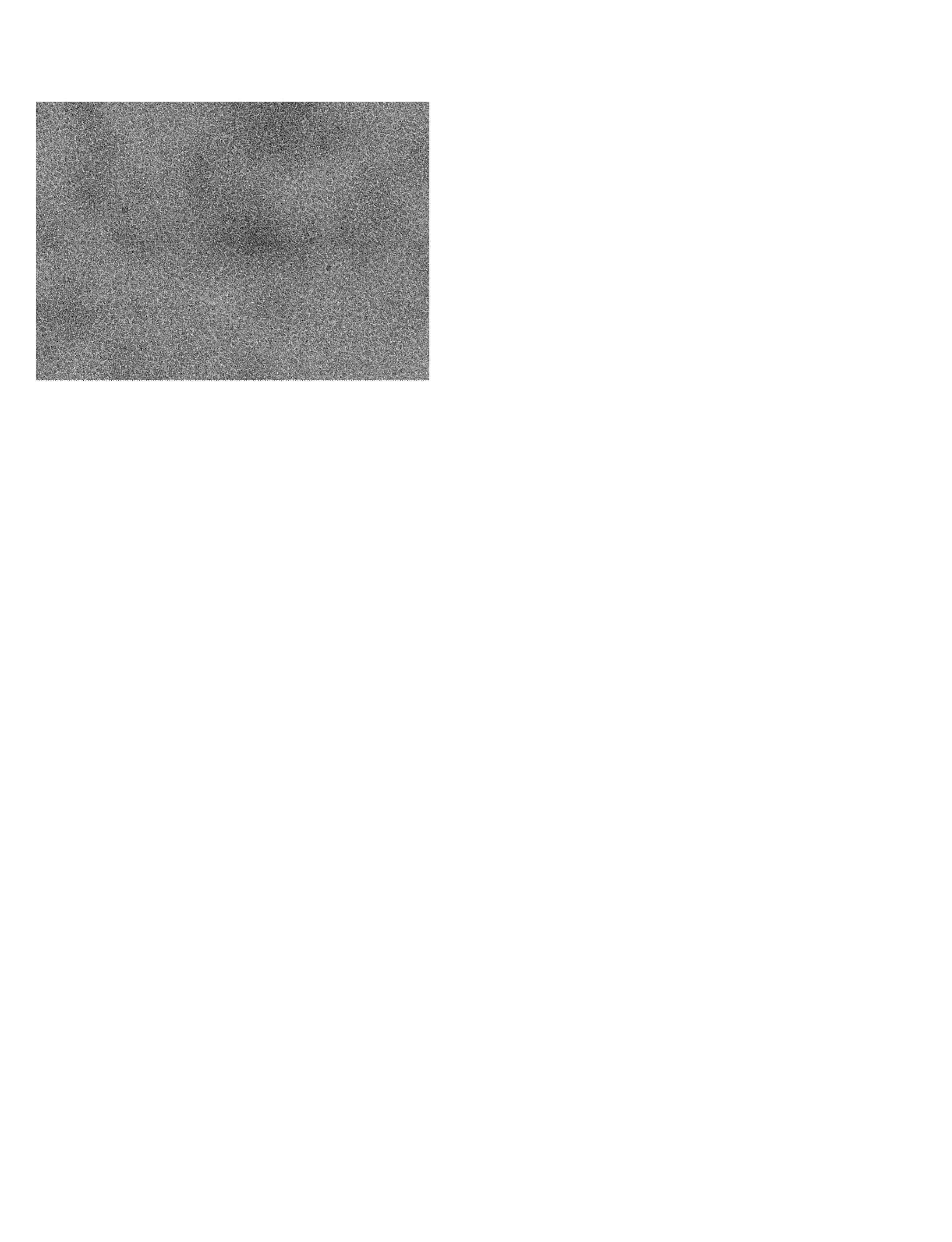

Supplement: Supplementary file 9 — Representative cryo-EM micrograph for CvHAS HA2-bound and UDP-bound dataset. [file 41594_2024_1389_MOESM9_ESM.jpg]
