# Supplementary material for: Structural insights into translocation and tailored synthesis of hyaluronan
Source: Nat Struct Mol Biol. 2024 Sep 25;32(1):161–71. doi: 10.1038/s41594-024-01389-1 (PMC11750622; doi:10.1038/s41594-024-01389-1)

Fig. 1c

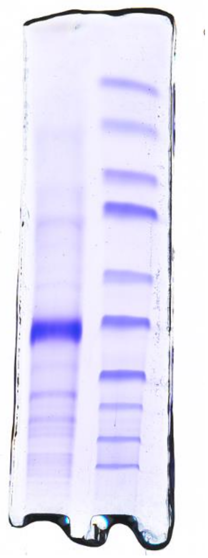

Fig. 1d,e

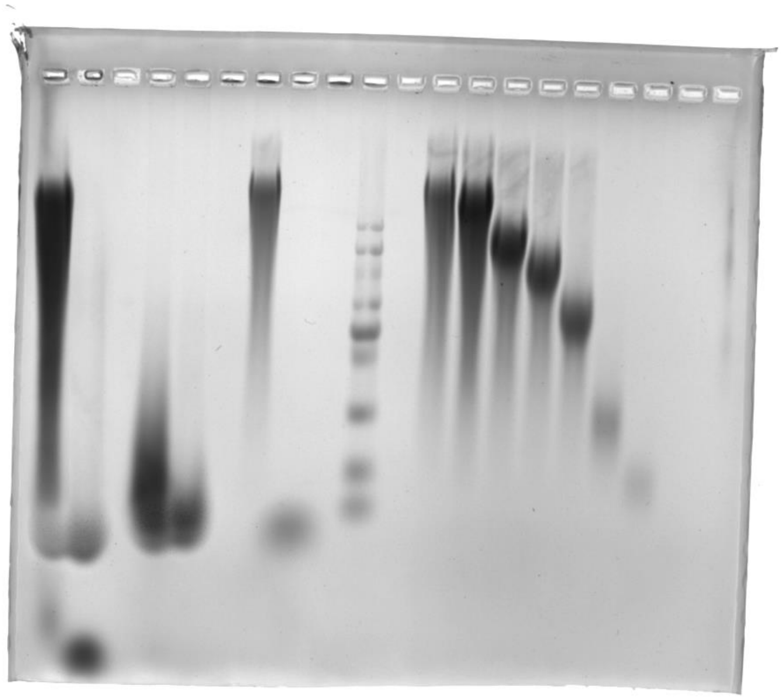

Fig. 1g

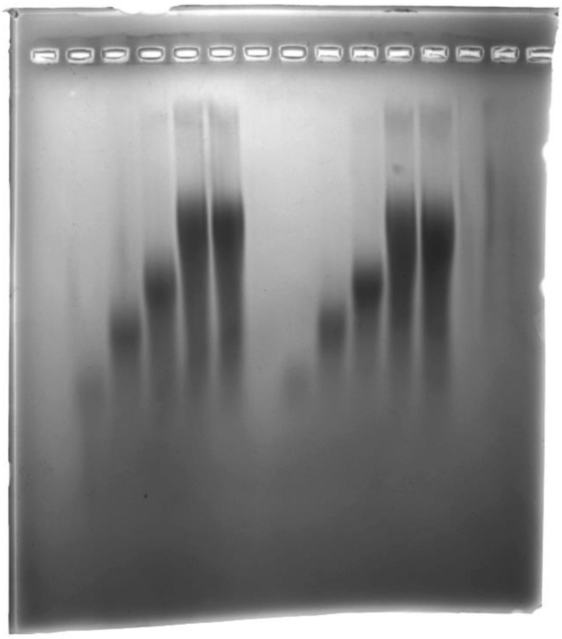

Fig. 1h

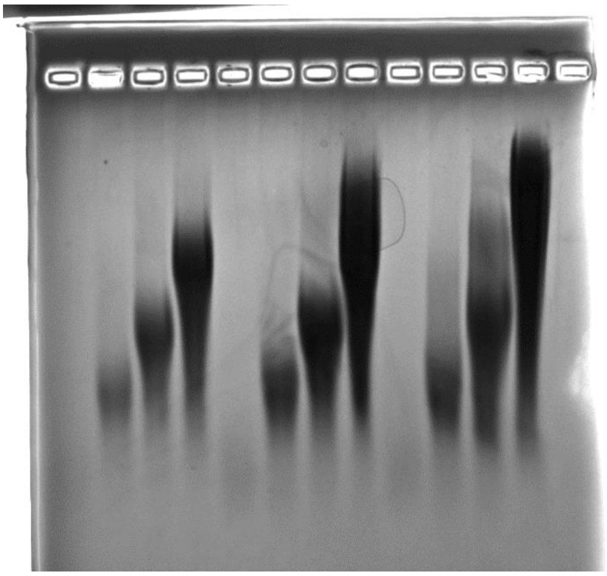

Fig. 4b short reaction

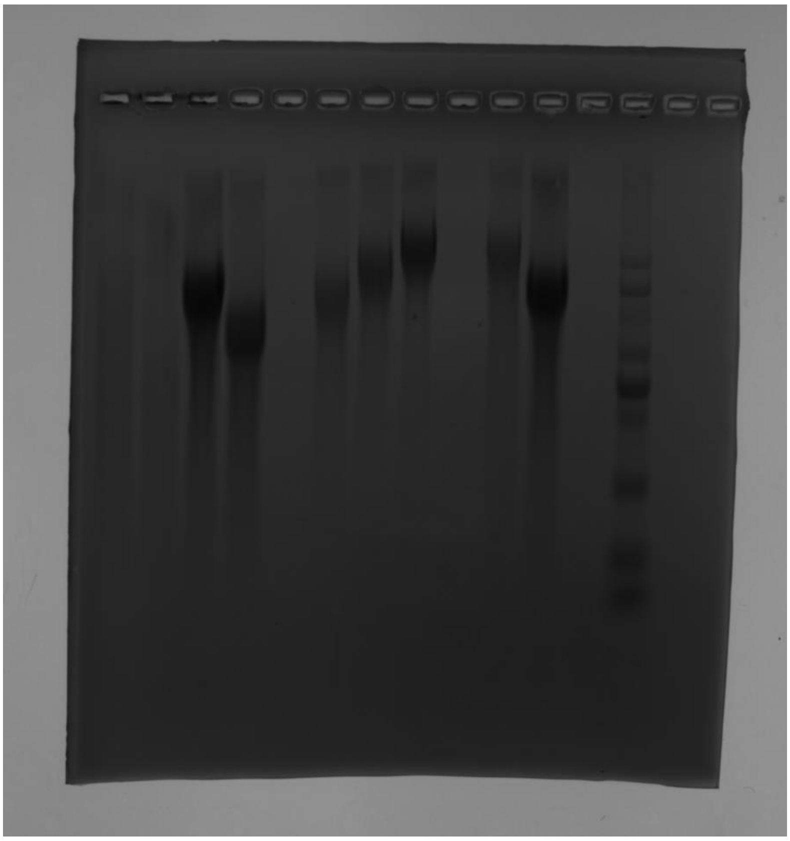

Fig. 4b long reaction, Fig. 4g

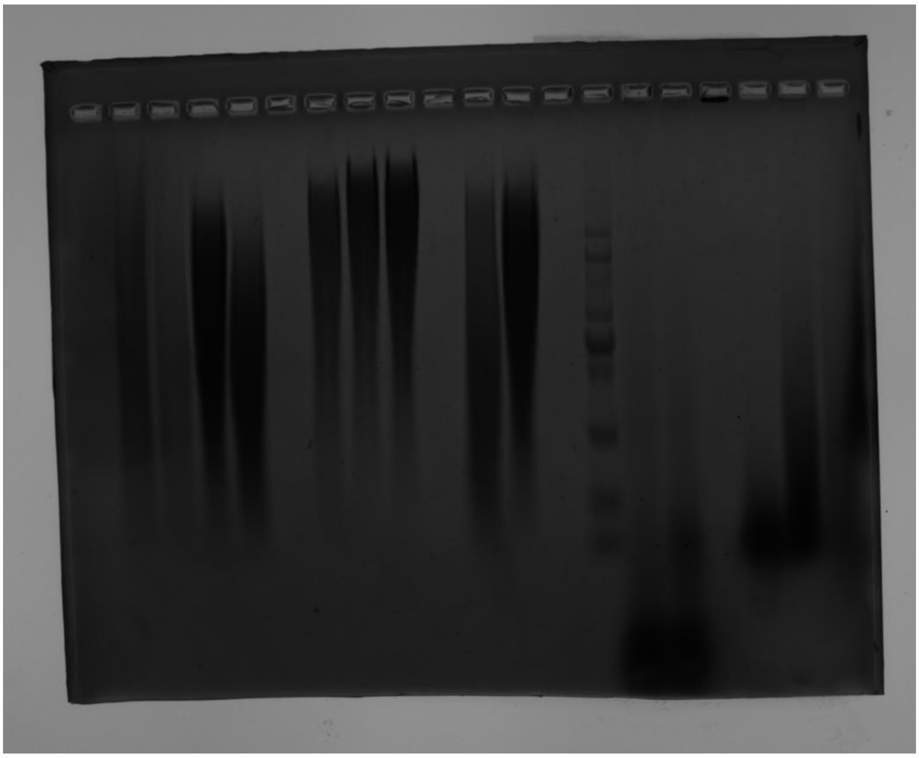

Fig. 4f short reaction

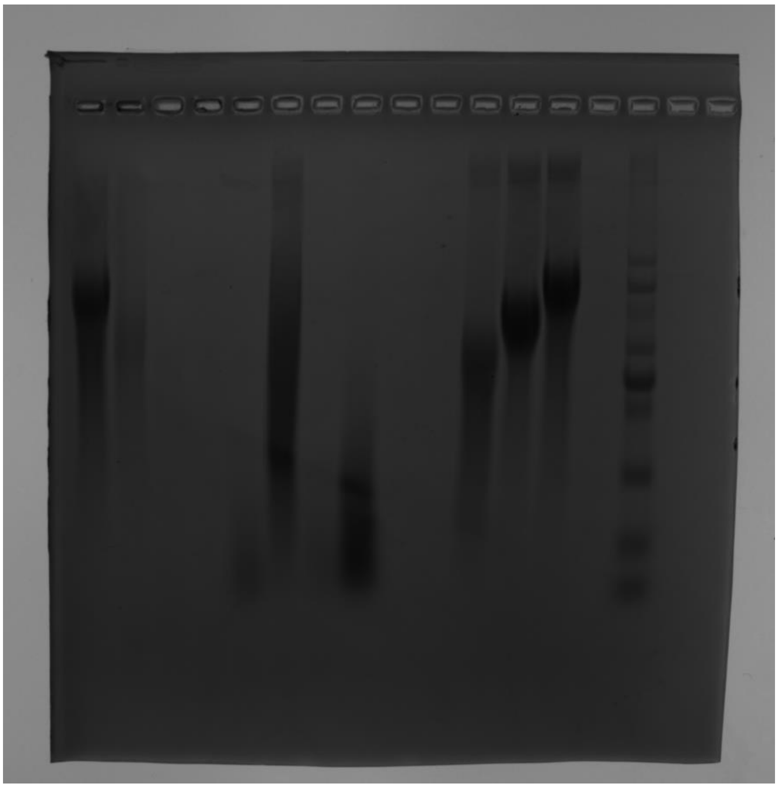

Fig. 4f long reaction

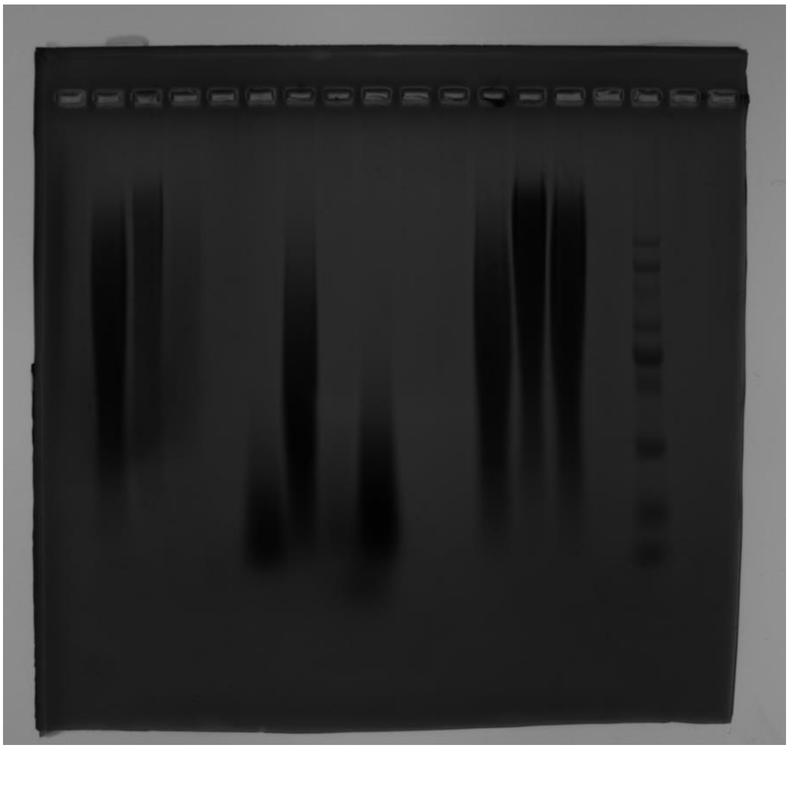

ED-Fig. 1a

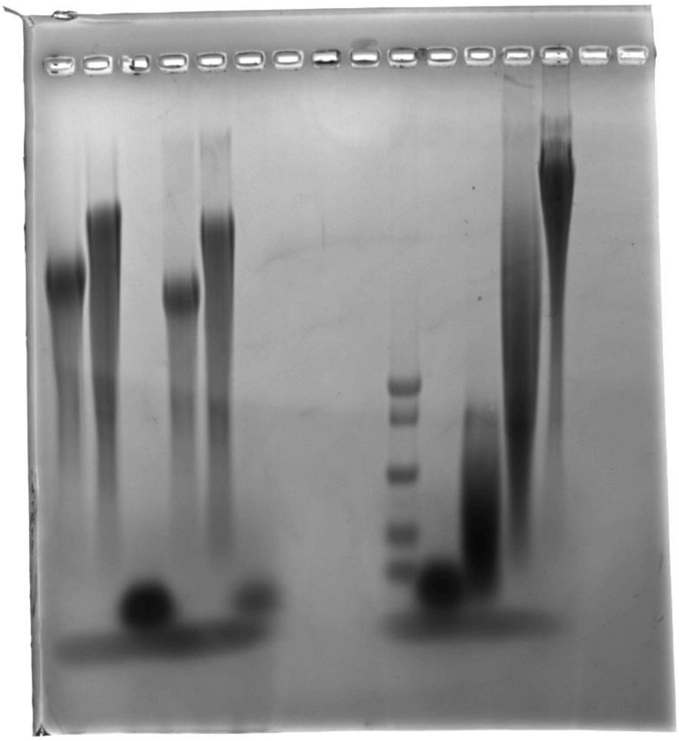

ED-Fig. 1d, ED-Fig. 1e

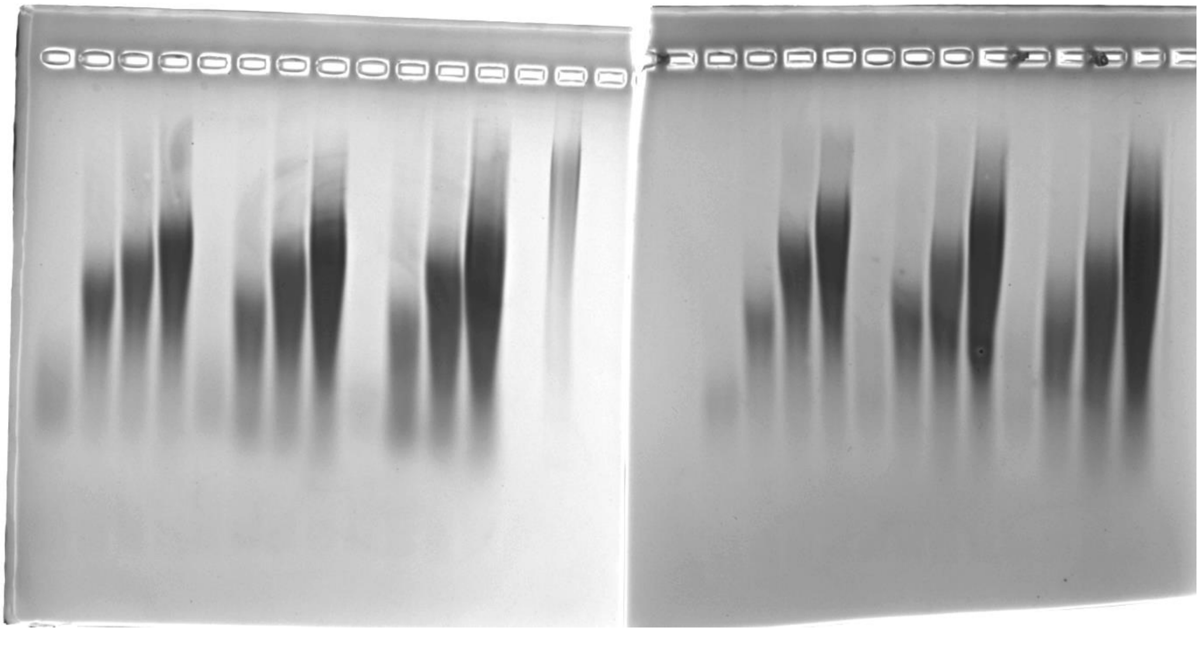

ED-Fig. 1f

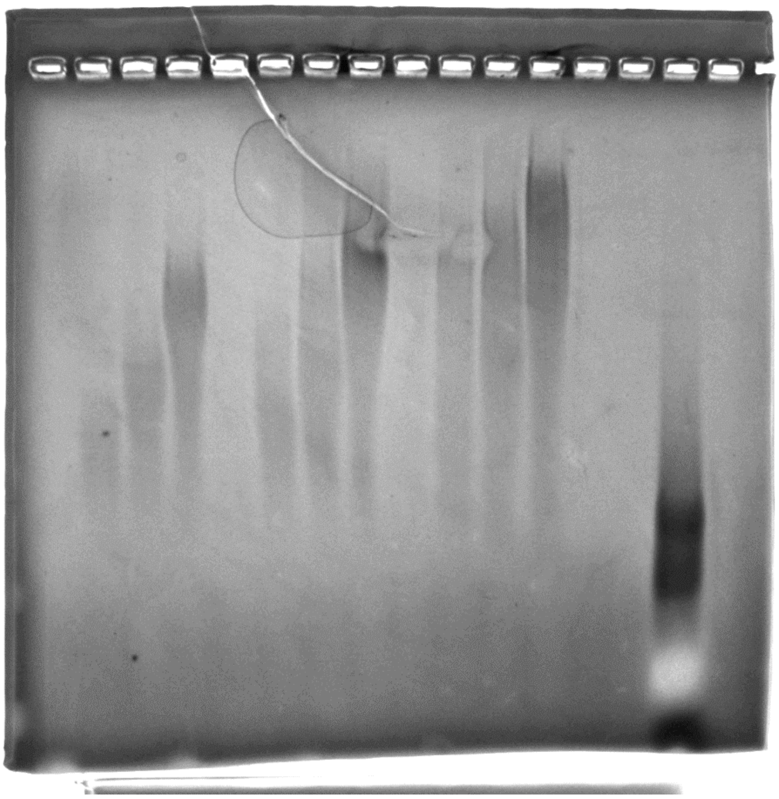

ED-Fig. 2e

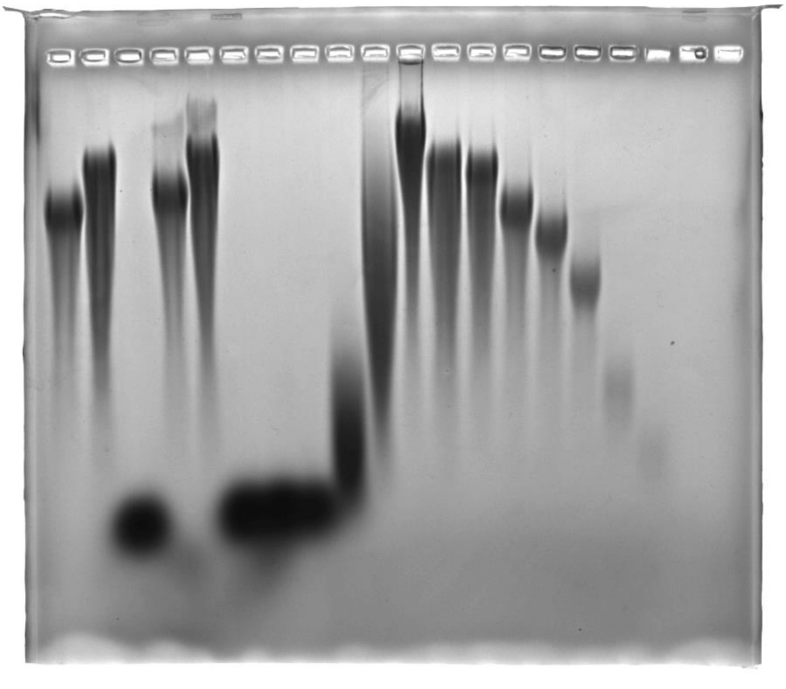

ED-Fig. 6e

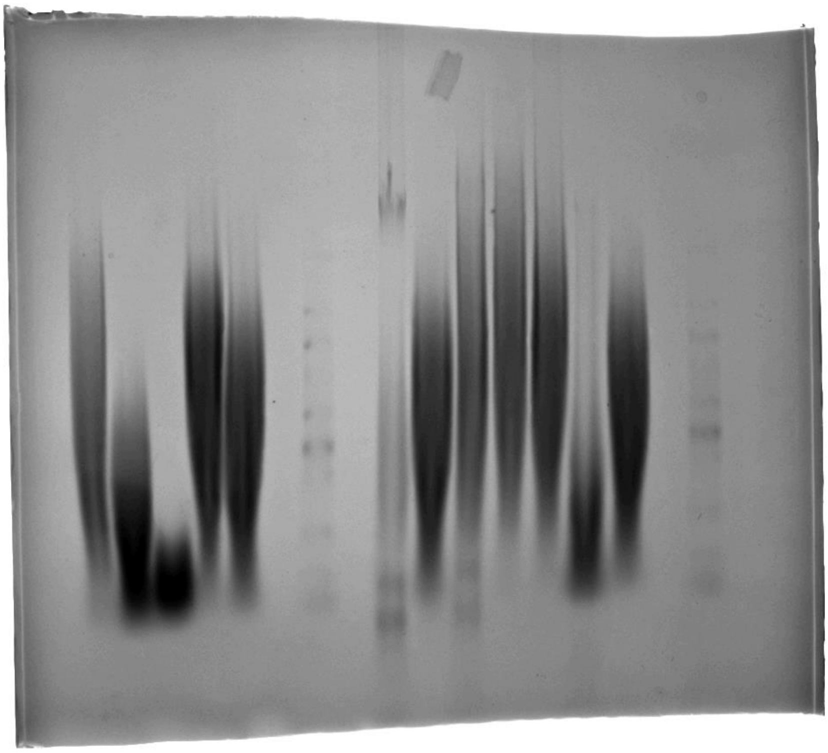

ED-Fig. 6f

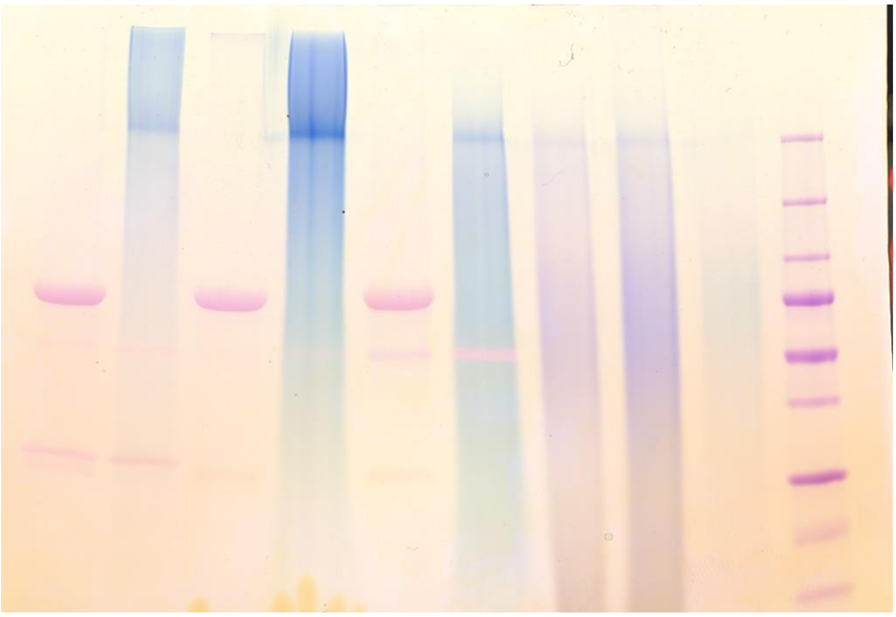

ED-Fig. 6g

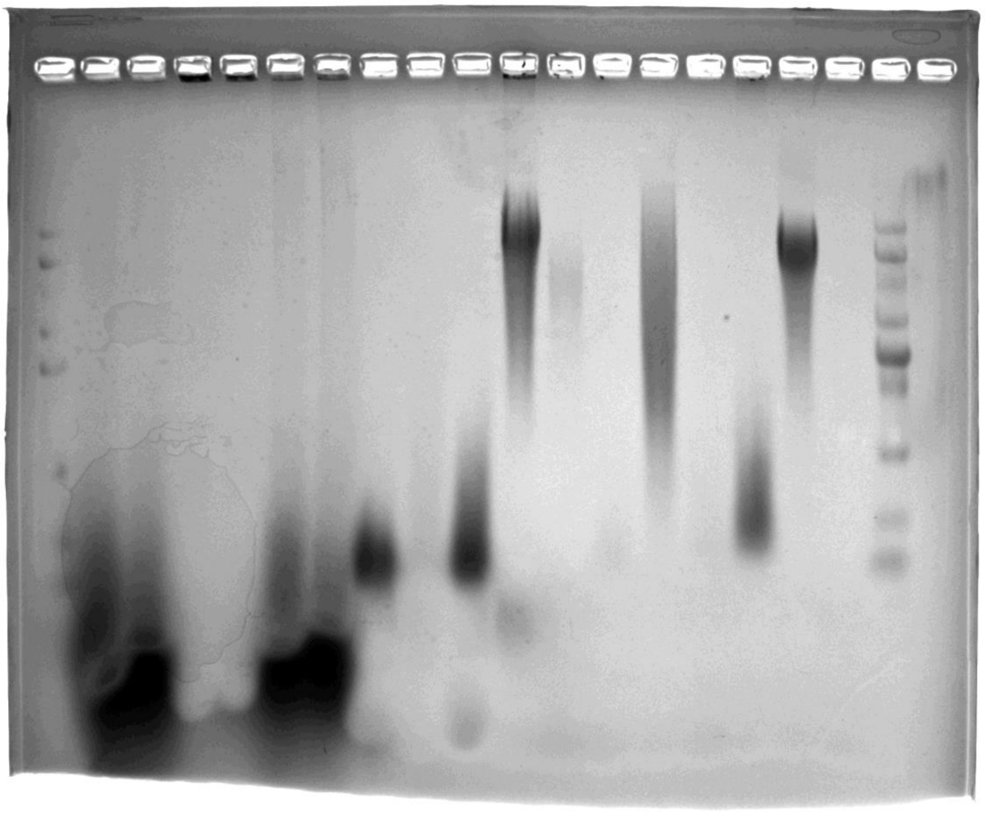

Supplement: Supplementary file 10 — Unprocessed gels, activity data and size-exclusion chromatogram data. [file 41594_2024_1389_MOESM10_ESM.pdf]
